# Challenges and Recommendations for the Deployment of Information and Communication Technology Solutions for Informal Caregivers: Scoping Review

**DOI:** 10.2196/20310

**Published:** 2020-07-29

**Authors:** Alhassan Yosri Ibrahim Hassan

**Affiliations:** 1 Centre for Socio-Economic Research on Ageing Italian National Institute of Health & Science on Ageing Ancona Italy; 2 Department of Economics and Social Sciences Faculty of Economics “Giorgio Fuà” Università Politecnica delle Marche Ancona Italy

**Keywords:** informal caregivers, ICT, digital health, eHealth, health economics, internet, health technology, ageing, home care, digital solutions

## Abstract

**Background:**

Information and communication technology (ICT)–based solutions have the potential to support informal caregivers in home care delivery. However, there are many challenges to the deployment of these solutions.

**Objective:**

The aim of this study was to review literature to explore the challenges of the deployment of ICT-based support solutions for informal caregivers and provide relevant recommendations on how to overcome these challenges.

**Methods:**

A scoping review methodology was used following the Arksey and O’Malley methodological framework to map the relevant literature. A search was conducted using PubMed, IEEE library, and Scopus. Publication screening and scrutiny were conducted following inclusion criteria based on inductive thematic analysis to gain insight into patterns of challenges rising from deploying ICT-based support solutions for informal caregivers. The analysis took place through an iterative process of combining, categorizing, summarizing, and comparing information across studies. Through this iterative process, relevant information was identified and coded under emergent broader themes as they pertain to each of the research questions.

**Results:**

The analysis identified 18 common challenges using a coding scheme grouping them under four thematic categories: technology-related, organizational, socioeconomic, and ethical challenges. These range from specific challenges related to the technological component of the ICT-based service such as design and usability of technology, to organizational challenges such as fragmentation of support solutions to socioeconomic challenges such as funding of technology and sustainability of solutions to ethical challenges around autonomy and privacy of data. For each identified challenge, recommendations were created on how to overcome it. The recommendations from this study can provide guidance for the deployment of ICT-based support solutions for informal caregivers.

**Conclusions:**

Despite a growing interest in the potential offered by ICT solutions for informal caregiving, diverse and overlapping challenges to their deployment still remain. Designers for ICTs for informal caregivers should follow participatory design and involve older informal caregivers in the design process as much as possible. A collaboration between designers and academic researchers is also needed to ensure ICT solutions are designed with the current empirical evidence in mind. Taking actions to build the digital skills of informal caregivers early in the caregiving process is crucial for optimal use of available ICT solutions. Moreover, the lack of awareness of the potential added-value and trust toward ICT-based support solutions requires strategies to raise awareness among all stakeholders—including policy makers, health care professionals, informal caregivers, and care recipients—about support opportunities offered by ICT. On the macro-level, policies to fund ICT solutions that have been shown to be effective at supporting and improving informal caregiver health outcomes via subsidies or other incentives should be considered.

## Introduction

The United Nations estimates that by 2050, 1 in 6 people in the world will be aged over 65 years, up from 1 in 11 in 2019 [1]. It is estimated that 1 in 3 older adults lives with more than a single chronic condition (eg, heart disease, diabetes, cancer, and dementia) [2,3], and this figure is closer to 3 out of 4 in older adults living in developed countries where it is predicted to rise significantly [4].

To manage chronic conditions more effectively, policy makers are supporting the idea of family-centered home-based care for older people rather than institutional care [5]. Older people are depending more on their families and friends for support with daily activities due to this shift from institutional to home care [6]. Informal caregivers are relatives, friends, and neighbors who care for older adults but are not trained or paid to provide care in contrast to formal caregivers, who offer paid professional services [7]. In 2013, the estimated economic value of unpaid informal care in the United States was $470 billion [8]. In Europe, 80% of all care is provided by informal caregivers, and estimates on the economic value of unpaid informal care in European Union member states range from 50% to 90% of the overall costs of formal long-term care provision [9]. Informal caregivers who care for old people save Canada’s health care system between $24 to $31 billion annually [10].

Lamura et al [11,12] pointed to the importance of novel technology solutions as a promising approach for empowering and supporting informal caregivers. Information and communication technology (ICT) consists of digital and analog technologies, including hardware, software, networks, and media, that facilitate collecting, capturing, storing, processing, transmitting, exchanging, and presenting information, and/or communication [13]. Barbabella et al [14] define ICT-based support solutions for informal caregivers as a service provided by any private or public organization that addresses caregiver and/or care recipient needs through technological devices that are integrated or not in a wider intervention program.

ICTs provide informal caregivers with remote access to information and training about caring-related issues though websites and online training materials [15]. They provide informal caregivers with personal support and social integration providing social, emotional, and peer support; social networking systems for peer support; and volunteer call networks [16]. Research showed that ICT solutions can improve psychological outcomes in informal caregivers [14-17]. ICT solutions may reduce caregiver depression, anxiety, stress, and burden as these solutions increase positive aspects of caregiving, caregiver self-efficacy, and confidence [14-17]. ICT solutions have also macro-level benefits as these solutions may help in the integration of informal and formal care through the reduction of inappropriate hospitalizations and lengths of stay. Consequently, the deployment of these solutions may generate savings contributing to the sustainability of the care systems [15-17].

Although ICT solutions can facilitate the delivery of home care and support informal caregivers of old people, there are many challenges to the deployment of these solutions [17]. Challenges are diverse and range from usability of technology solutions, sustainability, data security, digital literacy levels of informal caregivers, and other key issues [15-17]. Consequently, there is a need for mapping these different challenges and developing relevant recommendations on how to overcome them to inform the successful research and development of ICT solutions for informal caregivers. Although some reviews [18-21] have begun to synthesize the literature on ICT solutions for informal caregivers, they have focused solely on evaluating the effectiveness of these solutions and their impact on the informal caregivers, and the focus was mainly on informal caregivers of people with dementia. The aim of this scoping study is to provide an overview of the challenges of deployment of ICT-based support solutions delivered over the internet for informal caregivers of older people, which is, to the best of my knowledge, an unexplored field within the literature. Hence, this scoping review narrows the gap in the literature with respect to the ICT solutions designed for informal caregivers and the most frequently reported challenges for the deployment of these solutions. Moreover, by synthesizing the literature across the challenges of the deployment of ICT solutions delivered over the internet for informal caregivers of older people, this scoping review aims to provide relevant recommendations on how to overcome these challenges in order to guide future development of ICT solutions for informal caregivers.

## Methods

### Study Design

To carry out this scoping review, Arksey and O’Malley’s methodological framework [22,23] for conducting scoping reviews was followed. The five stages outlined by Arskey and O’Malley’s framework are as follows:

- Identification of the research question
- Identification of relevant studies
- Selection of relevant studies
- Charting the data from the selected literature
- Collating, summarizing, and reporting the results

The identified research topic includes a wide range of study designs addressing contexts in many countries as well as different technology solutions and a population of caregivers caring for older people with different chronic conditions. In order to comprehensively synthesize evidence to map this broad, complex, and emerging field of study, this framework has been selected as it is an appropriate approach to map a complex research topic and explore studies that use various methodologies, which is expected to be the case in this research topic [22,23]. Khalil et al [24] suggested using this inclusive approach in conducting scoping reviews to avoid potential exclusion of important information. The Preferred Reporting Items for Systematic Reviews and Meta-Analysis (PRISMA) guidelines have been followed (as far as relevant for a scoping review according to the PRISMA extension for scoping reviews) to verify the structure and content of this scoping review [25]. The checklist for the reported items according to the PRISMA extension for scoping reviews can be found in Multimedia Appendix 1.

### Stage 1: Identifying the Research Question

The research question for this scoping review was identified from a preliminary scan of the literature and meetings with different stakeholders representing national level caregiver organizations, researchers, and experts from the European Association Working for Carers (Eurocarers) also involving research centers working in these areas, the Centre for Socio-Economic Research on Ageing of the Italian National Institute of Health and Science on Ageing (IRCCS-INRCA), University Medical Center Groningen, and the Department of Economics and Social Sciences of Marche Polytechnic University. Due to the rapid technological change in recent years, there is a lack of consensus in the academic literature on the challenges faced by informal caregivers—and specifically those who care for dependent older adults living at home with chronic conditions—in using ICT-based support solutions. Hence, the following research questions have been developed for this scoping review: What are the challenges of the deployment of ICT-based support solutions delivered over the internet for informal caregivers of older people? What are the recommendations for overcoming these challenges?

### Stage 2: Identification of Relevant Studies

In order to capture the most relevant research studies in different domains (medical, engineering, social, economic, etc) on the challenges faced by informal caregivers of older people in using ICT solutions, the following databases were used to locate the relevant literature, as they contain relevant works in different domains: PubMed, IEEE library, and Scopus. Due to the rapid technological change in recent years, date restrictions were set in the period from January 1, 2015, to December 31, 2019, to capture recent and up-to-date relevant literature on ICT solutions. In order to address the components of the research questions, keywords and search terms were classified into four main groups:

- Keywords representing variations of the term informal caregiver (eg, informal carer, family caregiver)
- Keywords representing variations of the term older people (eg, old, aged)
- Keywords representing variations of the term challenges (eg, problems, barriers)
- Keywords of relevant ICT (eg, web, internet)

An overview of the different groups of keywords is presented in Table 1. Keywords were searched using Boolean operators. The search strategy can be found in Multimedia Appendix 2.

**Table 1:** Keywords and search terms.

Keywords	Search terms
Group 1	Family carers, informal caregivers, informal carers, family caregivers
Group 2	Old, elderly, aged, senior, older people
Group 3	Barriers, obstacles, challenges, problems, difficulties, complications, concerns
Group 4	Web, internet, online platform, information technology, mobile application, information and communications technology, ICT^a^

^a^ICT: information and communications technology.

### Stage 3: Selection of Relevant Studies for the Review

In order to best address the indicated research questions, inclusion and exclusion criteria have been identified.

The inclusion criteria were as follows:

- Publications in English language
- Studies with research aimed at investigating the use of ICT-based support solutions delivered over the internet, such as web platforms and mobile apps, for informal caregivers
- Literature focused on informal caregivers of dependent older individuals. The main focus of this scoping review is studies dealing with ICT solutions that address caregiver needs, relieve pressure on caregivers, and give remote access to information and training about caring-related issues
- Research studies using different methodologies (qualitative, quantitative, and systematic reviews) as well as theoretical papers

The following exclusion criteria were applied:

- Studies focused on informal caregivers of pediatric patients and disabled adults
- Studies focused on professional or paid caregivers
- Studies on other assistive technologies that are not delivered mainly over the internet (eg, assistive robots)
- Studies that took place in nursing homes or care facilities, as the main focus of this scoping study is the informal caregivers of older adults living at home

The database searches retrieved 454 studies for consideration. After reviewing further publications suggested by researchers and experts from Eurocarers, INRCA-IRCCS, University Medical Center Groningen, and the Department of Economics and Social Sciences of Marche Polytechnic University and reviewing the studies’ reference lists, an additional 6 studies were identified through these external sources. After removing duplicates, 359 studies remained.

**Figure 1:**
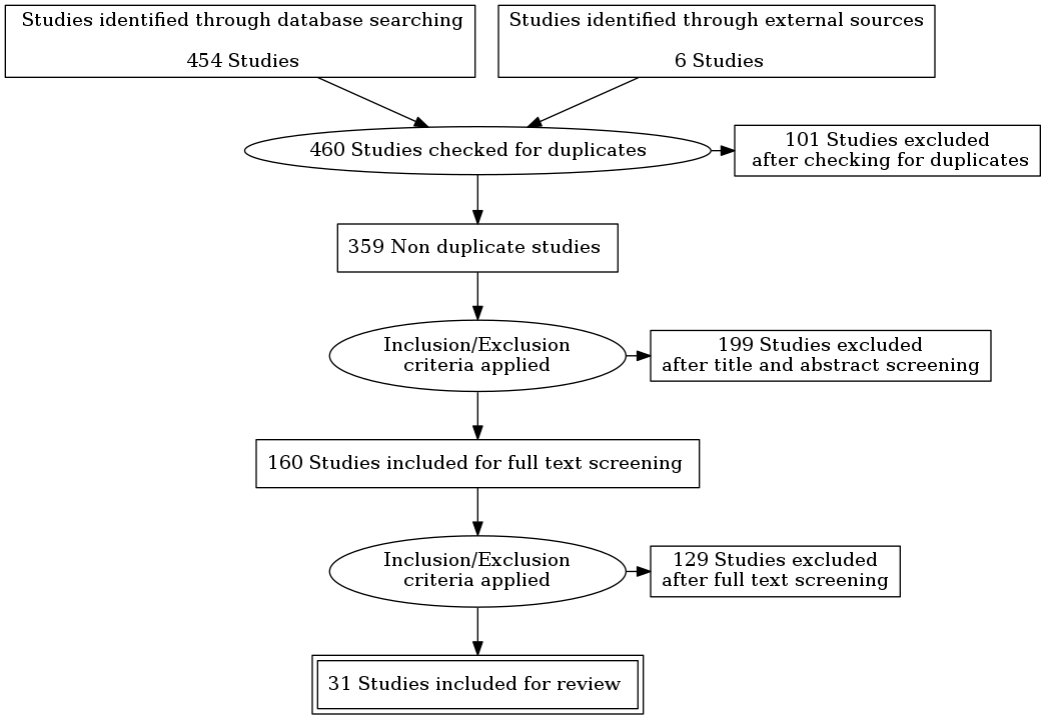
Study selection workflow.

Thereafter, a 2-stage process was followed. During the first one, the retrieved studies were screened by title and abstract to determine whether they met the selection criteria. In this first stage, 199 studies were excluded after title and abstract screening, and 160 publications identified for full text reading. In the second stage, 31 articles met the selection criteria and were included for the scoping analysis. In order to effectively manage the process of literature identification, citations obtained from the searches were imported into EndNote software (Clarivate Analytics) and Zotero software (Center for History and New Media at George Mason University) for reference management. Consequently, a master citation database was constructed to collate all the citations from various sources. Built-in functions of the software allowed duplicates to be easily detected. As consistent with the scoping review approach [22,23], the methodological quality of the published articles was not a selection criterion. This enabled the inclusion of a range of development, implementation, and evaluation studies using different methodologies. The overall study selection workflow is illustrated in Figure 1.

### Stage 4: Charting the Data

Data charts were created to organize information from the included studies. In line with Arksey and O’Malley’s methodological framework, data entered for each study included authors and publication year, study type and methodology, study location, study aim, condition of cared-for family member, caregiver sample, and relationship of the caregiver to the care recipient.

Out of the 31 studies included in this scoping review, 17 were qualitative studies, 4 were quantitative studies, 1 was a systematic literature review, 1 was an integrative literature review, and 8 used mixed methods. Of these 31 studies, 10 studies were conducted in the United States, 4 studies in Canada, 3 studies in the United Kingdom, 3 studies in the Netherlands, 3 studies in Sweden, 3 studies were international, 2 studies in Germany, and 1 each in Spain, Italy, Poland, Denmark, France, and Australia. A summary of the information for each included publication is shown in Table 2.

**Table 2:** Summary of the information for the included publications.

Authors	Year	Study type and methodology	Study location	Study aim	Condition of cared-for family member	Caregiver sample	Relationship of the caregiver to the care recipient^a^
Allemann et al [26]	2019	Qualitative study with focus groups	Sweden	Explore the perceptions of ICT^b^ solutions as supportive solutions among informal caregivers of persons with heart failure	Heart failure	23	Spouse/partner: 22; Child: 1
Andersson et al [27]	2017	Qualitative exploratory descriptive study based on semistructured in-depth interviews	Sweden	Describe working informal caregiver experiences of having access to the web-based family care support network	Different conditions	9	Spouse: 1; child: 7; in-law: 3; niece:1
Austrom et al [28]	2015	Qualitative longitudinal study with focus groups	US^c^	Assess the feasibility and acceptability of a web-based video support group offered in real time for informal caregivers of persons with dementia	Dementia	5	Spouse: 4; parent: 1; friend: 1
Barbabella et al [29]	2018	Mixed-methods sequential explanatory study with structured questionnaires and focus groups	Germany, Italy, and Sweden	Assess the use and usability of a psychosocial web-based program for informal caregivers	Different conditions	118	Spouse/partner: 34; child/child-in-law: 67; other: 17
Bergström and Hanson [30]	2018	Integrative literature review	International	Explore studies concerning ICT support of adult carers of older people	N/A^d^	N/A	N/A
Boessen et al [31]	2017	Mixed-methods study with semistructured interviews and questionnaire	The Netherlands	Test the usability and perceived value of an online platform that aims to support the communication and collaboration between informal and professional caregivers of patients with dementia	Dementia	7	Spouse/partner: 1; child: 6
Boots et al [32]	2016	Exploratory mixed-methods study with focus groups, interviews, and questionnaire	The Netherlands	Development and initial evaluation of a web-based support solution for informal caregivers	Early-stage dementia	28	Spouse: 22; child: 2; child-in-law: 2; sibling: 1; friend: 1
Coffey et al [33]	2017	Qualitative study with semistructured interviews	US	Identify preferred sources of health information for informal caregivers	Traumatic brain injury, spinal cord injury, or burn injury	32	Not reported
Cristancho et al [34]	2015	Mixed-methods unblinded monocentric pilot RCT^e^	France	Evaluate the efficacy and acceptability of a web-based psychoeducational program for informal caregivers of persons with Alzheimer disease	Alzheimer disease	49	Not reported
Dam et al [35]	2017	Qualitative study with semistructured interviews	The Netherlands	Test the development and feasibility of an online social support intervention for informal caregivers of people with dementia	Dementia	23	Not reported
Duggleby et al [36]	2019	Mixed-methods secondary analysis study	Canada	Compare users and nonusers of a web-based intervention for informal caregivers of older people	Alzheimer Disease	92	Not reported
Gaugler et al [37]	2016	Mixed-methods study with survey and semistructured interview	US	Test the feasibility of an online resource for dementia caregivers	Dementia	30	Not reported
Gibson et al [38]	2015	Qualitative study with semistructured interviews	UK^f^	Explore the everyday use of ICT by people with dementia and their families	Dementia	26	Not reported
Grossman et al [39]	2018	Quantitative content analysis study	International	Identify mobile apps geared toward caregivers of older adults, catalog features, and suggest best practices for adoption	N/A	N/A	Not reported
Heynsbergh et al [40]	2018	Qualitative study with focus groups and semistructured interviews	Australia	Understand how digital technology may be used to address informal caregiver needs	Cancer	45	Spouse: 29; parent: 13; other (relative/friend): 3
Holden et al [41]	2018	Qualitative study with semistructured interviews	US	Understand the current personal health information management practices in informal caregiving for adults with and without dementia	Dementia	10	Not Reported
Kales et al [42]	2017	Qualitative study with focus groups	US	Develop a caregiver-focused, web-based program to assess and manage behavioral and psychological symptoms of dementia	Dementia	26	Adult child: 15; spouse: 6; other relative: 5
Kim [43]	2015	Quantitative cross-sectional and descriptive correlational design study using a secondary analysis	US	Understand internet use among dementia informal caregivers	Dementia	450	Spouse: 29; parent: 15; child or grandchild: 335; other type of relative: 38; friend/nonrelative/neighbor: 29; missing data :4
Núñez et al [44]	2016	Pilot randomized controlled pre-post	Denmark, Poland, and Spain	Assess the satisfaction of the informal caregivers with an ICT platform	Dementia	61	Not reported
O’Connor et al [45]	2016	Qualitative exploratory study with focus groups and interviews	UK	Explore barriers experienced by participants during the co-design of mobile app for informal caregivers	Dementia	16	Not reported
Phongtankuel et al [46]	2018	Qualitative study with semistructured interviews	US	Explore informal caregiver receptivity and concerns in using mHealth apps	Different conditions	80	Child: 46; spouse:10; other relative: 19; friend: 5
Ploeg et al [47]	2018	Qualitative study with semistructured interviews	Canada	Understand how web-based support help informal caregivers	Multiple chronic conditions	56	Spouse/partner: 31; son/daughter :22; daughter-in-law: 2; grand-daughter: 1
Schaller et al [48]	2016	Mixed-method design with questionnaires and interviews	Germany	Assess the usefulness and impact of the eHealth Monitor Dementia Portal service in the dementia care	Dementia	25	Spouse:11; child: 9; relative: 5; grand-daughter: 1
Schulz et al [49]	2016	Quantitative study with online survey	US	Assess whether and how much informal caregivers are willing to pay for technologies designed to help monitor and support care recipients	Different conditions	512	Not reported
Sriram et al [50]	2019	Systematic review	International	Explore the positive and negative aspects, knowledge, acceptance, and ethical issues in the use of assistive technology by caregivers of persons with dementia	N/A	N/A	N/A
Tonsaker et al [51]	2016	Qualitative study with focus groups	Canada	Investigate how caregivers access and use information on the internet about caregiving and their perspectives on the design and features of a new personal health experiences website	Different conditions	16	Not reported
Turk et al [52]	2019	Qualitative study with focus groups and semistructured interviews	UK	Explore the perceived usefulness and ease of use of a personalized web-based resource for informal caregivers	Different conditions	50	Not reported
Vaughan et al [53]	2018	Mixed-methods study with survey, focus groups and semistructured interview	US	Examine use and perceptions of a web-based social support intervention for informal caregivers	Different conditions	211	Spouse/partner: 186; other: 31
Walker et al [54]	2016	Qualitative study with focus groups	US	Gain insights into how older people and their families manage health information and communication	Different conditions	23	Not reported
Werner et al [55]	2017	Qualitative study with focus groups	US	Identify barriers to information needs of informal caregivers to manage dementia-related behavioral symptoms	Dementia	26	Not reported

^a^Some participants in some studies cared for multiple family members. Therefore, counts do not add up to the sample size.

^b^ICT: information and communication technology.

^c^US: United States.

^d^N/A: not applicable.

^e^RCT: randomized controlled trial.

^f^UK: United Kingdom.

### Stage 5: Collating, Summarizing, and Reporting the Results

NVivo software version 12 (QSR International) was used to collect and organize study data. Full articles were imported as pdf files into NVivo software program for data extraction, analysis, and coding. The analysis took place through an iterative process of combining, categorizing, summarizing, and comparing information across studies. Through this iterative process, relevant information was identified and coded under emergent broader themes as it pertained to each of the research questions. Inductive thematic analysis was conducted as described by Braun and Clarke [56] to analyze the texts from the included studies. Patterns of challenges of the deployment of ICT solutions delivered over the internet for informal caregivers of older people were filtered out from an initial literature search and the previous meetings with experts in the field to develop an optimal category system. If challenges were found that did not fit into any previously known pattern, categories were iteratively added [23,57]. For each identified challenge, a list of recommendations on how to overcome it was created based on the findings of this scoping analysis and the suggestions provided by the reviewed studies as a guide for future development, research, and innovation in the area of ICT solutions for informal caregivers.

## Results

### Emerging Themes

This section provides a detailed description of the emerging themes around the challenges influencing the deployment of ICT solutions for informal caregivers that rose from the analysis of the included studies. According to the analyzed studies, four major categories of challenges could be identified:

- Technology-related challenges
- Organizational challenges
- Socioeconomic challenges
- Ethical challenges

Table 3 provides an overview of the identified challenges, showing the prevailing theme and the possible co-themes for each challenge. The categories were used to organize identified challenges as commonly presented in the studies reviewed. Under each individual theme, the challenges were ordered from the least overlapping (ie, most specific to the relevant theme) to the most overlapping (ie, least specific to the relevant theme). Due to the complexity of ICT and the interconnectedness of the identified challenges, a nonoverlapping categorization of individual identified challenges was not possible. In order to optimize the classification system, the categories were refined through several reiterations of the revision process. Each of the identified challenges was assigned to the most prevailing theme and additional possible co-themes. For instance, the challenge privacy came under the prevailing theme ethical challenges, as the abuse of data leads to violation of ethical principles, but it also fell into the additional possible co-theme technology-related challenges, as ICT solutions need security authentication measures and strong encryption mechanisms.

**Table 3:** Overview of the identified challenges with the prevailing theme and the possible co-themes for each challenge.

Challenge	Type of challenge			
	Technology-related	Organizational	Socioeconomic	Ethical
Design and usability of technology	x^a^			
ICT^b^ solutions are time-consuming	x			
ICT solutions lack specificity	x			
ICT solutions are not integrated in different devices	x			
ICT solutions don’t provide diverse content	x			
Distrust in technology	x	y^c^		
Digital illiteracy	x	y	y	y
ICT may replace other support measures for informal caregivers		x		
Lack of awareness		x		
Lack of Interoperability and fragmentation of support solutions	y	x		
Gap between research and ICT-based support solutions providers	y	x		
Funding and cost of technology		y	x	
Sustainability and lack of business models		y	x	
ICT may create inequality			x	y
Poor ICT infrastructure	y	y	x	
Autonomy				x
Privacy	y			x
Technophobia and dehumanization of care	y	y		x

^a^x: prevailing type of challenge.

^b^ICT: information and communication technology.

^c^y: overlapping with additional possible co-themes.

In the following, a brief description is provided for each of the subdimensions identified for each macro-challenge.

### Theme 1: Technology-Related Challenges

The progress that has been taking place in developing ICT solutions for informal caregivers has been widely recognized [15-21]. However, many informal caregivers find it challenging to incorporate such technologies into their daily work routines because of the struggles experienced with the technological component of the ICT-based solutions (eg, the difficulty in using technology solutions developed for informal caregivers and their poor design). This theme provides an overview of the identified challenges related to this issue.

#### Design and Usability of Technology

In the literature reviewed, informal caregivers reported technical troubles related to the design and usability of the solutions [31,35,39,44]. With regard to the former, the design process plays an important role in ICT use, acting as a barrier or a facilitator [39]. Many informal caregivers experience difficulties in navigating ICT solutions, due to the complexity of the design of technology [58]. This is partly due to the fact that informal caregivers are not included in the design of ICT-based support solutions even though these solutions should be co-designed with end users [58]. Gélinas-Bronsard et al [58] reported that this type of design may constrain informal caregiver behavior accordingly and inadequately reflect their needs as users of ICT solutions.

As for the second issue, many informal caregivers report challenges related to the usability of the solutions. They include aspects such as navigation through the menus [44], help and search options [44], high battery consumption of some apps on smartphones [31], insufficient instructions to participants regarding the use of the different functionalities of the solutions [31], and complex log-in procedures [35]. The inefficiency deriving from the difficulties experienced by caregivers due to either design- or usability-related issues decreases the overall ease of using ICT solutions [35].

#### Information and Communication Technology Solutions Are Time-Consuming

In the reviewed literature, informal caregivers find ICT-based supporting solutions to be time-consuming [33,39,43,44]. This time consumption may overload the caregiving daily routines instead of supporting them and thus increases the burden [36]. Furthermore, informal caregivers are a group of people for whom time is typically strained, hence they prefer simple and easy-to-operate technologies [39]. When caregivers spend longer times providing direct caregiving tasks, they do not have the time or energy to search for information and seek support via the internet [33,39,43,44].

Coffey et al [33] mentioned that informal caregivers have very little extra time to participate in online activities such as calls and webinars and they avoid spending too much time using complicated technologies. This is in line with the findings of another reviewed study by Nunez et al [44] where informal caregivers found the supporting solution in the study to be time-consuming given the solution could not be integrated with their existing medical records, which was perceived as a complication by them.

#### Information and Communication Technology Solutions Lack Specificity

Technology solutions are not optimized to specific informal caregiver needs as it could be hard to recognize themselves in the information provided through ICT [26]. The content delivered via ICT solutions is often static and not specific to caregiving needs [34,41]. The literature reported that the experience of seeking information about caregiving situations using ICT solutions appeared to be a chaotic and confusing process, as informal caregivers need to access multiple information routes to get the information they need [34,37,38,55]. In many studies, informal caregivers reported difficulties in finding specific and up-to-date information related to their caregiving situation which adapts to changing needs over time [41,48,55].

#### Information and Communication Technology Solutions Are Not Integrated in Different Devices

Some ICT services are only available via single channel (eg, web) although informal caregivers might use other devices (eg, tablets, smartphones) [35,52]. Turk et al [52] reported that many informal caregivers use devices other than computers to access online services such as tablets and smartphones to overcome some of the extrinsic hardware barriers to accessing the service.

#### Information and Communication Technology Solutions Do Not Provide Diverse Content

The content delivered on many ICT solutions is basic (eg, only textual content rather than video content) [26,52]. ICT-based support solutions lack visual graphics such as video content, which is necessary to mitigate verbose text and help informal caregivers with language barriers to access the content [52].

#### Distrust in Technology

Some informal caregivers don’t feel confident about the information provided via ICT solutions [41,55]. Informal caregivers reported their concerns about the quality of the content presented via ICT solution [33,34,51]. The content is almost superficial, and finding credible concrete information for informal caregivers still proved to be a challenging task [33,34]. Some studies report that some informal caregivers avoided networking via ICTs and sharing experiences with other informal caregivers who were previously unknown to them as they prefer to have previous knowledge of potential peer contacts [27,41].

#### Digital Illiteracy

Digital literacy is a set of skills associated with the use of ICT that every individual should develop to be able to perform in a computerized society [59]. Many informal caregivers are middle-aged to older people with a low to middle educational level [16], and these sociodemographic characteristics are often associated with a low level of digital skills, which could prevent them from benefiting from ICT [39].

The literature identifies digital illiteracy as a major challenge for informal caregivers as it increases the need for additional time and effort to adopt a new technology [26-29,36,44,52,58]. Lack of digital skills is found to be a main barrier for informal caregivers in using ICT solutions in many studies analyzed in this review [26,28,29,31,37,48]. For example, as reported in a study by Barbabella et al [29], informal caregivers in their study sometimes avoid chatting and networking due to experiencing the ICT-based support solutions as technically challenging for them as they have insufficient digital skills. Boessen et al [31] reported that the limited use of ICT solution in their study was due to a mismatch between informal caregiver digital competence and technology requirements of the solution.

### Theme 2: Organizational Challenges

There is a common concern in the literature [26,27,31,43,52,54,58] that there are many organizational gaps in terms of the fragmentation of ICT-based support solutions for informal caregivers, lack of coordination between statutory support solutions for informal caregivers, and lack of awareness of the availability of these solutions. This theme highlights some of these organizational challenges that fall between the micro- and macro-level.

#### Information and Communication Technology May Replace Other Support Measures for Informal Caregivers

Informal caregivers fear that ICT support solutions might substitute other statutory forms of support that they are already receiving, thus ending up reducing the overall support at their disposal [52]. It was noted that informal caregivers may be hesitant to adopt ICT-based support solutions unless they are assured that these solutions will not affect their rights in other statutory support as respite care, access to training, and recognition of skills and work-life balance measures [43,52].

#### Lack of Awareness

Informal caregivers perceive a lack of relevant information on the available ICT-based support solutions and their benefits. Policy makers, health care professionals, and other stakeholders lack awareness and/or are unconvinced of the opportunities that ICTs bring to all the actors involved in home care provision [38,52,58]. Lack of awareness of ICT solutions available to informal caregivers means that informal caregivers are unlikely to access these solutions, and this could have an impact on the number or severity of unmet support needs [40]. The issue of the lack of awareness of ICTs may be particularly pertinent to older informal caregivers [52]. This imbalance suggests that greater efforts need to be made to reach this population and show them the value of this technology for their specific needs [39].

#### Lack of Interoperability and Fragmentation of Support Solutions

Interoperability is the ability of different information systems, devices, and apps (systems) to access, exchange, integrate, and cooperatively use data in a coordinated manner, within and across organizational, regional, and national boundaries to provide timely and seamless portability of information and optimize the health of individuals and populations globally [60]. Some countries lack standards and regulations regarding the deployment of ICT in health care which leads to fragmentation of services, and many ICT solutions for informal caregivers lack the ability to exchange and make use of information among systems and software [31,54,58]. The lack of interoperability between the digital health information systems in many countries and the ICT-based support solutions is a challenge for the deployment of ICT solutions, as the inability to integrate different support solutions for informal caregivers entails double recording of information which limits the use of these solutions [31,54,58].

#### Gap Between Research and Information and Communication Technology Solutions Providers

The reviewed literature acknowledges that research that assesses the use of technology for informal caregivers is still based mainly on pilot programs, and further assessment of these programs is needed to understand the main findings and possibly enlarge their scope of application [26,27,29,39,51]. Moreover, most of the informal caregiving ICT solutions currently on the market do not seem to have been developed with the guidance of caregiving researchers [39]. Therefore, there may be gaps between ICT solution features and empirical findings regarding informal caregiver effective means of intervention [39].

There is also a knowledge gap with regard to how informal caregivers perceive ICT in their everyday life in relation to these needs [26]. The insight into how ICT is perceived by informal caregivers is important knowledge that could be used in developing interventions to support informal caregivers as well as supporting the implementation of ICT [26,39]. Andersson et al [27] argued that the paucity of research exploring informal caregiver experiences increases the risk of implementing ICT solutions that do not address the actual support preferences, concerns, and perceptions of the informal caregiver. This gap may discourage informal caregivers from using ICT solutions [26,27].

### Theme 3: Socioeconomic Challenges

The literature identifies a series of socioeconomic issues that might have a negative influence on the deployment of ICT-based support solutions delivered over the internet for informal caregivers of older people. Questions such as who should fund ICT solutions for informal caregivers and whether a successful business model can be demonstrated for these solutions are common in the literature [26,36,38,42,44,49,52,58]. In the following, some of the challenges related to this category of issues are highlighted.

#### Funding and Cost of Technology

Lack of financial availability to invest in the acquisition of technologies is perceived as a barrier by informal caregivers. They tend to believe that governments should pay for the delivery and deployment of technology solutions [49]. Informal caregivers also indicate that waiting lists for publicly funded programs significantly delay the access and procurement of ICT solutions [38,58].

#### Sustainability and Lack of Business Models

The lack of business models and evidence on the long-term impact and sustainability of ICT solutions beyond trials and pilots leads to considerable variations in the perceived role and importance of such technologies from the point of view of users and health care providers as well as policy makers [30,42,49,58].

Gélinas-Bronsard et al [58] argued that sustainability is a main challenge to the deployment of the ICT solutions, and that this depends on technological, organizational, and political factors. Hence, obtaining government support for caregiving technologies will likely require strong evidence of successful and sustainable business cases [49]. On the other hand, few ICT solutions have been translated into a deliverable and sustainable service [42]. Further studies operating at a macro-level are therefore essential to develop more rigorous proofs about the sustainability of ICT solutions [30].

#### Information and Communication Technologies May Create Inequality

Informal caregivers from lower socioeconomic backgrounds may be less able to use ICT-based support solutions, which might lead to health inequality [58]. Moreover, the literature identifies a potential social justice issue if governments do not value these support solutions and a “user-pays” implementation—a pricing approach based on the idea that the most efficient allocation of resources occurs when consumers pay the full cost of the goods that they consume—must be used for ICT solutions. This would then limit the accessibility of the solutions to only those who can afford to pay [26,44,52,58].

#### Poor Information and Communication Technology Infrastructure

The lack of adequate ICT infrastructure in some countries acts as a barrier to the deployment of ICT for informal caregivers. Poor connectivity to the internet, particularly for informal caregivers in rural areas, is a barrier to the use of any ICT solutions delivered over the internet [36,58]. Poor ICT infrastructure and underperforming internet connection is also a concern for research with ICT solutions and possibly an exclusion criterion for participants in efficacy and effectiveness trials [36].

### Theme 4: Ethical Challenges

The use of ICT solutions raises ethical concerns as issues of privacy and autonomy, among other moral issues, can be present. Questions about how ICT solutions are dehumanizing the care process, how the privacy of informal caregivers and their care recipients is protected, or the confidentiality of the information provided are raised throughout the literature [29,32,34,35,39,41,46,53].

#### Autonomy

Ethical issues around who held the power of choice of use and discontinuance of ICT solutions arise from some studies [41,50,54]. Informal caregivers have concerns on controlling care recipients via ICT and on who has the power of choice of use and discontinuance of ICT [50]. This concern is related to the fear that constant monitoring of care recipients via ICT solutions is restricting their freedom [50]. There is a consensus among informal caregivers that care recipients must be involved as much as possible in the selection and use of ICTs [41].

#### Privacy

Informal caregivers have concerns on data ownership and privacy of the data [27]. Grossmann et al [39] point out that privacy concerns may be especially relevant to older informal caregivers, who voice the most concerns over the privacy and security of their information online. Literature shows that informal caregivers are concerned that if they have uploaded personal information this would compromise their safety and there might be the possibility of other people reading and accessing their private notes [27,52]. Concerns about online privacy and confidentiality are reported in many studies [42,46,48,49,52,58], showing that informal caregivers are often afraid that their personal data may be misused and manipulated.

#### Technophobia and Dehumanization of Care

Many informal caregivers are described as having technophobia, which is partially explained by the human contact that usually characterizes caregiving tasks and the fear that technology introduction could disrupt the caregiving nature [16]. Some informal caregivers are concerned that ICT solutions would replace the personal component of caring [50]. ICTs are perceived by informal caregivers as being too impersonal and entailing limited personal interaction and individualization, which could cause mistrust of information and cause a feeling of ICT solutions being less usable [26]. For some informal caregivers, it is considered important to have personal contact with the health care personnel to obtain individualized information [26,58]. The literature notes that informal caregivers view ICT solutions as beneficial in terms of helping with care, but less preferable to care provided by a real person [28,36,38,40]. Many studies reported the lack of desire of the informal caregiver to engage with ICT solutions because of the impersonal nature and their preference to access support using methods such as face-to-face settings [40,47,53].

### Recommendations for Overcoming Challenges of the Deployment of Information and Communication Technology Solutions for Informal Caregivers

The emerging themes obtained during this scoping analysis allowed the extraction of insights that were grouped to create a series of recommendations for overcoming the challenges of deployment of ICT solutions based on the suggestions from the reviewed studies. Table 4 presents, for each identified challenge, recommendations to overcome this challenge based on the findings of this scoping analysis.

**Table 4:** Recommendations to overcome each of the identified challenges.

Challenge	Recommendations to address or overcome the related challenge
Design and usability of technology	Implement user-centered and participatory design strategies to improve usability [55] Implement features that only add value for informal caregivers and avoid designing extra services of which not all are necessary [31] Designers should involve older informal caregivers in the design process as much as possible as opposed to simply testing with younger demographics [39,50] Designers should follow a design process that integrates feedback loops and adaptations based on specific needs of informal caregivers that may change over time [26,47,55]
ICT^a^ solutions are time-consuming	Combine as many useful features as possible into easy-to-use solutions in order to reduce informal caregiver load to help their productivity rather than hinder it [39] Design care coordination tools in solutions to allow better coordination between multiple informal caregivers in providing care [37]
ICT solutions lack specificity	Content introduced on ICT solutions for informal caregivers should be personalized, tailored, and specific to informal caregiver individual situations. Feedback loops should be integrated in the different solutions to improve tailoring and allow the content to be adaptive to changing needs over time [26,38,42,48,55] Health and social care professionals should be consulted by ICT solutions designers when introducing content for informal caregivers to change the focus of these tools from providing general information to providing more specific information [27] ICT solutions should provide dynamic, flexible, and more customizable content based on a structure that favors interaction with professionals and peers, such as online community support. They should facilitate creating new templates for information specific to the diagnosis and caregiving needs of the care recipient. This includes the ability to select or filter situationally pertinent information [26,34,41] Informal caregivers should be able to compile information incrementally through the disease progression so as to not become overwhelmed [41] ICT solutions for informal caregivers should match the needs of the informal caregivers, rather than informal caregivers being molded to match what ICT-based support solutions are available for them [50] Dedicated virtual desks or online appointments through ICT solutions with informal caregivers would be helpful for getting more tailored health information, personalized advice, and counseling on clinical aspects of the care recipient [29]
ICT solutions are not integrated in different devices	ICT solutions should be integrated in different devices, not only web-based. Web platforms should be optimized for use on devices other than computers such as tablets and smartphones [31,52]
ICT solutions don’t provide diverse content	Content delivered via ICT solutions should be diverse and not only textual. Video content including more visual graphics is important to mitigate verbose text and associated language barriers [26,52] ICT solutions should provide content at accessible levels, reducing the use of complicated medical language and adjusting literacy levels by providing content at different knowledge levels [26,47,55]
Distrust in technology	Solutions should use trusted information sources with evidence-based materials and provide citations for sources of information [55] Training informal caregivers on how to evaluate ICT solutions is important to improve informal caregiver confidence in accessing quality information via support solutions [33] Features that combine the utility of the internet with the expertise of medical professionals, including care-support hotlines, have been shown to improve informal caregiver trust in technology solutions [33]
Digital illiteracy	Actions that build informal caregiver technical and computer skills early in the caregiving process are important for optimal use of available ICT-based support solutions [27,28] Assessment of informal caregiver needs and digital skills is essential to educate and support informal caregivers on how to operate ICT solutions [26,44,52] Training and technical support would need to be an ongoing activity and not a one-off task [29,58] ICT solutions should also include educational programs to increase computer literacy with illustrated features embedded into the solutions to assist informal caregivers who have low computer literacy [36] ICT solutions should be easy to use by accommodating a range of informal caregiver skills and abilities [41,42,53]
ICT may replace other support measures for informal caregivers	ICT-based support solutions should be tailored in a coordinated way with other existing services such as respite care, access to training, and recognition of skills and work-life balance measures [9,43,52]
Lack of awareness	Strategies are needed to raise awareness among all stakeholders, including policymakers, health care professionals, and informal caregivers and care recipients, about all support opportunities afforded by ICT [29,51] Health professionals should consider providing informal caregivers with information on ICT solutions available to them as a means of additional support and guide them in terms of selecting solutions with evidence-based content [39]
Lack of interoperability and fragmentation of support solutions	Defining standards and regulations on interoperability of information and devices and enhancing integration with existing ICT systems in health and social care is necessary [41,54] Integrating disparate systems rather than adding content to the multiple solutions one already uses (eg, calendar, personal health record, educational materials) [41] Designing services for informal caregivers and care recipients that improve interoperability through interfaces that connect and communicate across institution-specific portals. Improving interoperability allows connecting informal caregivers and care recipients with multiple providers in the most care recipient–centered manner [54]
Gap between research and ICT solutions providers	Future developers should collaborate with academic researchers to ensure that their solutions are designed with the current empirical evidence in mind [39] User experience studies are needed to customize ICT solutions to the needs, desires, and abilities of the informal caregivers [39,40] Future development of ICT solutions should consider a theory-based approach and how to best meet the complex transition-related needs of informal caregivers [45,47] Research is needed to better understand the impact of ICT solutions for informal caregivers when used in combination with other forms of support, including professional and peer support [47]
Funding and cost of technology	Policy makers and insurance providers should consider policies promoting the use of ICT solutions that have been shown to be effective at supporting and improving informal caregiver health outcomes via subsidies or other incentives [39]
Sustainability and lack of business models	ICT solutions must be offered early in the caregiving process, and its support functions need to be adaptable over the course of the caring trajectory [27] Identification of sustainable business models, exchange of good practices, collection of evidence, and transferability of optimal solutions among localities, regions, and countries are all important to continue allocating public funding for initiatives [49]
ICT may create inequality	Promote digital inclusion policies, providing access and promoting the use of ICT solutions for informal caregivers [26,44,52] Foster continuous development of digital competencies in informal caregivers [29,58]
Poor ICT infrastructure	Governments and policy makers should allocate funding for improving ICT and digital infrastructures [39,43,49]
Autonomy	Informal caregivers should be given the choice to accept or refuse access to ICT solutions. Access to all ICT solutions should be regulated by the primary informal caregiver and the care recipient [41] Care recipients should be involved as much as possible in the selection and use of ICT solutions [50]
Privacy	Involving municipal family care advisors known to the users of ICT solutions in the administration of the tools enhances experiencing ICT as safe and secure [27] ICT solutions need to focus on standard development guidelines and security authentication measures such as passwords, strong encryption mechanisms, and informative privacy policies [39,46]
Technophobia and dehumanization of care	Blending online support with regular face-to-face support can increase the acceptance of ICT solutions [32] Involving different health care professionals in the provision of professional support leads to overcoming possible skepticism and lack of knowledge about ICT solutions [29,32,34] Guidance by professional moderators or volunteers might provide practical hands-on advice to informal caregivers and increase their engagement with ICT solutions [35]

^a^ICT: information and communication technology.

Table 5 provides an overview of the prevailing perspective and additional possible perspectives for intervention for each challenge. This overview aims to identify at which level possible solutions to each specific challenge might take place. Solutions may take place at the following levels: system (macro), provider (meso), and user (micro). For instance, possible intervention perspectives for the challenge “lack of awareness” may be at the user level, as informal caregivers are unlikely to access these solutions without awareness and information on the available ICT solutions and their benefits, but they could also be at the system and provider levels, also needed to raise awareness among stakeholders, including policy makers and health care professionals, about support opportunities afforded by ICT.

**Table 5:** Overview of the prevailing and possible additional levels of interventions recommended to address the identified challenges.

Challenge	Level of intervention		
	System (macro)	Provider (meso)	User (micro)
Design and usability of technology	y^a^	x^b^	y
ICT^c^ solutions are time-consuming		y	x
ICT solutions lack specificity		x	
ICT solutions are not integrated in different devices		x	
ICT solutions don’t provide diverse content		x	
Distrust in technology			x
Digital illiteracy			x
ICT may replace other support measures for informal caregivers	y	y	x
Lack of awareness	y	y	x
Lack of interoperability and fragmentation of support solutions	x	y	
Gap between research and ICT solutions providers	x	y	
Funding and cost of technology	x	y	
Sustainability and lack of business models	y	x	
ICT may create inequality	y	y	x
Poor ICT infrastructure	x		
Autonomy		y	x
Privacy		y	x
Technophobia and dehumanization of care	y	y	x

^a^y: additional possible perspectives for intervention.

^b^x: prevailing perspective for intervention.

^c^ICT: information and communication technology.

## Discussion

### Principal Findings

The purpose of this scoping review was to identify challenges related to deployment of ICT solutions delivered over the internet for informal caregivers of older people. Emerging themes for these challenges were divided and classified for better understanding. These insights were used to produce a series of recommendations for overcoming the challenges of deployment of ICT solutions delivered over the internet for informal caregivers of older people. The recommendations will contribute richly to future ICT developments for informal caregivers and for this rapidly growing technological context.

### Challenges of Deployment of Information and Communication Technology Solutions for Informal Caregivers

The findings highlighted in this review demonstrate that deployment of ICT solutions delivered over the internet for informal caregivers of older people is coming with a variety of challenges. These range from specific challenges related to the technological component of the ICT-based service regarding design and usability of technology to organizational challenges such as fragmentation of support solutions to socioeconomic challenges such as funding of technology and sustainability of solutions to ethical challenges around autonomy and privacy of data. These findings confirm previous studies performed in the field and integrate them with a conceptually grounded classification system for organizing the different challenges into four specific thematic categories. Other studies have also highlighted the challenges of deployment of ICTs for informal caregivers [11,12,15,16]. Kluzer et al [15] mentioned that older informal caregivers with low or no digital skills have difficulties using ICT solutions and lack of a sustainable business model in ICTs is one of the major challenges of their deployment. Cucculelli et al [61] raised the importance of sustainability models in ICTs and digital innovation. The lack of successful business models in the case of ICT solutions for informal caregivers was one of the major challenges to the deployment of ICTs for informal caregivers in the European Commission’s policy report on technology-based services support and long-term care challenges in home care [16]. One explanation for that could be the economic value of ICTs as support service for informal caregivers is not always a direct translation of the value found in other areas of activity where a sustainable business model could be identified [17].

The analysis reveals that challenges to deployment of ICT solutions delivered over the internet for informal caregivers of older people are diverse and overlap at the same time. The challenges of deployment of these solutions, although reported as separate issues in the literature, appear to be at least partially interrelated. For instance, the digital illiteracy of many informal caregivers means it takes more time to use ICTs so that, with caregiving demands, less time is available. Moreover, ICT-based support solutions are perceived by informal caregivers as being too impersonal causing distrust in these solutions, which leads in turn to a feeling that ICT solutions might be less usable.

### Recommendations and Implications

Based on the categories of challenges and the overlap existing between the elements of challenges in each theme, recommendations were extrapolated. As shown in Table 4, each challenge on its own can suggest a series of relevant recommendations. The recommendations relevant to each specific challenge might take place at different levels of intervention.

With regard to the challenges related to the technological component of the ICT-based service, the main recommendations were to follow participatory design and involve older informal caregivers in the design process as much as possible as opposed to testing with younger users. Technology solutions that are user-friendly for younger adults may not be user-friendly for older users. This is in line with other studies demonstrating that participatory design increases the adoption of technology solutions and their use by the intended users [62]. Designers need to identify how technologies are incorporated into the everyday lives of informal caregivers and care recipients [30]. Designing care coordination tools in solutions is important to allow better coordination between multiple informal caregivers in providing care, which may introduce ICT-based support solutions as time-saving solutions rather than time-consuming tools [37]. Contents introduced in ICT solutions for informal caregivers should be personalized, tailored, and specific to individual situations of informal caregivers. In this context, feedback loops should be integrated in the different solutions to improve tailoring and allow the content to be adaptive to changing needs over time. Additionally, information delivered through ICT could cause added stress if not carefully worded. Therefore, when producing content via ICT solutions, questions such as whom it will reach and how the information could affect those reading it need to be properly addressed. Adapting information to the relationship the informal caregiver has to the care recipient is important, too [26].

An example of an ICT solution that responds to the challenges mentioned is a solution such as a web platform or mobile app that offers interactive tools allowing specific information to be provided to informal caregivers. The design of the solution should be interactive with feedback loops allowing immediate response to the needs of the informal caregivers, aiding in the decision process and selection of the most relevant information [63]. Content should be developed with a focus on the basic needs of daily life and supported by demonstration videos of procedures and audio documents [63]. The design must be intuitively based and should not require prior knowledge [64]. As many informal caregivers are middle-aged to older people, the text view should have a font size compatible with the characteristic limitations of old age and allow the user to adjust it depending on the device and the viewing distance [65]. The information should be clear and simple providing alternative text information with animation, video, and audio. Decreasing the number of pages informal caregivers must access is recommended in a solution, and consequently the workload, probability of errors, and time needed for using the solution will be reduced. Validation processes in accessing accounts that involve issues such as asking the user about a significant date should be avoided as that may be confusing and demotivating for older informal caregivers.

Another important recommendation in this theme addresses the suggestion to undertake actions on a large scale to equip informal caregivers with the needed digital skills. Lamura et al [12] highlighted the importance of improving older informal caregiver digital literacy based on an early assessment of their needs and digital skills. Research showed that older adults are capable of learning and acquiring digital literacy skills as long as they know the functional benefits related to ICT [59]. Thus, this is in line with another recommendation in this theme to combine the utility of the internet with the expertise of medical professionals, including care-support hotlines, to improve informal caregiver trust in technology solutions and their functional benefits.

This issue is also connected with the recommendations formulated to address and overcome organizational challenges. Health care professionals should consider providing informal caregivers with information on ICT-based support solutions available to them and guide them in terms of selecting the most appropriate solutions. In a study analyzing 12 of the most relevant ICT-based initiatives in Europe to support informal caregivers of older people living in the community [16], raising awareness of the opportunities provided by ICT-based services for informal caregivers among all stakeholders has been identified as one of the main policy recommendations. Public information campaigns are needed to increase knowledge about support opportunities afforded by ICT for informal caregivers. At the macro-level, defining standards and regulations regarding the deployment of ICT in health care is important to overcome the fragmentation of ICT solutions [41,54].

Furthermore, health information systems should start addressing how best to get information from care recipients and their informal caregivers into the systems. It is important to support bidirectional conversations instead of focusing only on getting information from health care systems out to care recipients and their informal caregivers. The cooperation of informal caregivers with information about their care recipients is required for health care professionals to be successful in their roles and impart appropriate professional advice [66]. The interaction of health care professionals and all parties with an interest in supporting informal caregivers such as caregiver advocacy organizations with informal caregivers becomes an integral part of the value chain that supports both communication and coordination. Hence, these parties should all be more engaged with developing ICT solutions to link them with informal caregivers.

Among the topics emerging in the socioeconomic challenges theme, one of the most frequently reported difficulties in the reviewed studies is the availability of adequate funding of ICT solutions and their sustainability over time. Governments should consider policies to fund ICT solutions that have been shown to be effective at supporting and improving informal caregiver health outcomes via subsidies or other incentives. In this context, it is crucial to promote an exchange of effective practices, collection of evidence, and transferability of optimal solutions among localities, regions, and countries, and across different bodies and organizations in order to optimize the deployment of ICT solutions [17]. Furthermore, ICT solutions must be offered early in the caregiving process, and its support functions need to be adaptable over the course of the caring trajectory [27]. Governments and policy makers should allocate funding for improving ICT and digital infrastructures. On the other hand, even though ICT-based support services have the potential to play a role in supporting caregivers, they are not likely, by themselves, to be a complete solution. To alleviate the burden and isolation that many informal caregivers experience, governments and policy makers should consider that other social support systems are needed for informal caregivers. ICT solutions should be tailored in a coordinated way with other existing services as respite care, access to training, and recognition of skills and work-life balance measures.

Finally, with regard to the ethical challenges associated with the deployment of ICT solutions, this review recommends that ICT solutions should be sensitive to informal caregiver privacy concerns and the extent to which a technology might undermine their autonomy, control, and dignity. In this context, blending online support with involving health care professionals in the provision of professional support leads to overcoming possible skepticism. Previous studies related to ICT solutions for informal caregivers have shown that caregivers embrace the connection with health care professionals to support them in their caregiving, and this connection also enhances experiencing ICTs as safe and secure solutions [67,68].

### Limitations

Some limitations concerning this review need to be considered. First, a quality assessment of the methodology adopted by the selected papers was not used to exclude publications, and results from studies using a variety of study designs and author opinions were incorporated into the findings of this review. However, this is in line with the principles usually applied by the scoping review methodology, and all the publications included in this review are peer reviewed. Another limitation is that only one researcher conducted the data extraction and the analysis for this review. Hence, there is a potential that some data were not extracted. Also, a systematization of all the challenges of the deployment of ICT solutions for informal caregivers without any overlaps was not possible due to the complexity of ICTs. Although the highest possible quality standards for classification were followed in an iterative process, possible limitations in the analytical approach should be taken into account when interpreting the results. Moreover, considering recent developments in technology solutions, the classification of challenges may not cover all contexts where technology solutions (eg, assistive robots) are used on a minute-by-minute basis, and this may pose a different set of challenges. Furthermore, although a strict scoping review framework was followed, there is a chance that relevant research may have been omitted, especially when searching a large body of evidence produced in the form of non-English publications and grey literature.

### Conclusions

Despite a growing interest in the potential offered by ICT solutions for informal caregiving, diverse and overlapping challenges to their deployment still remain. The deployment of ICT solutions for informal caregivers is accompanied by technology-related challenges such as the complexity of technology solutions and their poor design, which decrease the overall ease of using ICT solutions. Designers for ICTs for informal caregivers should follow participatory design and involve older informal caregivers in the design process as much as possible. A collaboration between designers and academic researchers is also needed to ensure ICT solutions are designed with the current empirical evidence in mind. In many studies analyzed in this review, a lack of digital skills has been found to be a main challenge for informal caregivers in using ICTs. The study concluded that taking actions to build informal caregiver digital skills early in the caregiving process is crucial for optimal use of available ICT solutions. Moreover, the lack of awareness of the potential added value and trust toward ICT-based support solutions requires strategies to raise awareness among all stakeholders—including policy makers, health care professionals, informal caregivers, and care recipients—about all support opportunities offered by ICT. Another frequently repeated challenge in the reviewed studies is the funding of ICT solutions and sustainability. On the macro-level, policies to fund ICT solutions that have been shown to be effective at supporting and improving informal caregiver health outcomes via subsidies or other incentives should be considered. Ethical issues such as dehumanization of the care process by ICT solutions and privacy protection of informal caregivers and their care recipients are also often reported throughout the literature. Informal caregivers should be given the choice to accept or refuse access to ICT solutions. In this regard, there is a necessity to involve health care professionals and municipal family care advisors known to the users of ICT in the administration of these tools in order to enhance the experience of ICT as a set of safe and secure tools.

The recommendations from this study can provide guidance and assistance for the deployment of ICT-based support solutions for informal caregivers, filling a gap in the currently available knowledge. Nevertheless, due to rapid technological innovation, more research needs to be conducted and guidelines for designing and developing ICT solutions should be made adaptable to continuous change, as new tools become available and health care delivery systems experience a technologically supported transition toward home care. More research is needed to measure the prevalence of using ICT as a source of care-related information among informal caregivers. Moreover, it is important to determine whether certain characteristics of informal caregivers such as their gender, relationship to the care recipient (eg, spouse/partner, child, friend), health status, or socioeconomic status appear to make a difference in their use of ICT to obtain care-related information. Understanding the sociodemographic and socioeconomic profiles of informal caregivers could help improve the quality of ICT solutions and tools by producing age- or gender-specific online information platforms. On the macro-level, further studies are necessary to ascertain the availability, efficiency, and sustainability of ICT-based support solutions. Research on impacts at these levels should be collected to complement those at user level and to convince policy makers to promote policy frameworks for the creation of ICT-based support solutions for informal caregivers.

## Appendix

Multimedia Appendix 1 Preferred Reporting Items for Systematic Reviews and Meta-Analyses extension for scoping reviews checklist.

Multimedia Appendix 2 Search strategy.

## Abbreviations

ICT: information and communication technology
IRCCS-INRCA: Centre for Socio-Economic Research on Ageing of the Italian National Institute of Health and Science on Ageing
PRISMA: Preferred Reporting Items for Systematic Reviews and Meta-Analysis
