# Supplementary material for: Challenges and Recommendations for the Deployment of Information and Communication Technology Solutions for Informal Caregivers: Scoping Review
Source: JMIR Aging. 2020 Jul 29;3(2):e20310. doi: 10.2196/20310 (PMC7424480; doi:10.2196/20310)
Supplement: Multimedia Appendix 2 [file aging_v3i2e20310_app2.docx]

## Multimedia Appendix 2

The following search strategy strings have been constructed using the disjunction “OR” logical operator between individual keywords within each group of keywords and the conjunction “AND” logical operator between groups of search terms:

((((((((((web) OR Internet) OR online platform) OR information technology) OR mobile application) OR (Information and communications technology)) OR ICT)) AND (((((((barriers) OR obstacles) OR challenges) OR problems) OR difficulties) OR complications) OR concerns)) AND (((((old) OR elderly) OR older people) OR aged) OR senior)) AND (((((((Family Carers) OR informal caregivers) OR informal carers) OR family caregivers))) AND ("2015/01/01"[PDat]: "2019/12/31"[PDat])).
